# Surgical Aspects of Spinal Tumours

**Published:** 1920-01-31

**Authors:** 


### SURGICAL ASPECTS OF SPINAL TUMOURS.

### A Classical Clinical Case.

In a recent clinical lecture on the "Surgical Aspects of Spinal Tumours," Mr. Percy Sargent, C.M.G., D.S.O., F.R.C.S., describes a most interesting case, which marked a new epoch in spinal surgery.

In 1881 the late Mr. Frederick Page, a surgeon practising in Newcastle, wrote as follows: "The operation of trephining the spine, proposed many years ago and adopted several times, has made no progress in surgery; nor is it likely to do so. It is an operation not within the range of practical surgery."

But alas! for so positive a prophecy, it was only a few years later that Sir Victor Horsley, in 1887, deliberately opened the spinal canal and removed a tumour which existed between the membranes and the cord.

### A Pioneer Operation.

This classical case, which marks the real beginning of operative interference in the region of the spinal column, may be briefly recalled. The patient was operated upon by Horsley after a diagnosis of "tumour pressing on the cord" had been made by Sir William Gowers, of University College Hospital, in June 1887. The patient was forty-two years of age, and had suffered for three years from a peculiar pain localised to a spot beneath the lower part of the left scapula. Some months before the operation, power was gradually lost in both lower limbs, and later urinary retention and impairment of cutaneous sensation was complained of. The symptoms culminated in a complete paraphlegia, with loss of all forms of sensibility up to the level of the ensiform cartilage. Just above this level there was severe girdle pain, worse on the left side than the right, and increased to agony on any movement.

### A Successful Result.

At the operation Sir Victor Horsley removed the arches of the upper thoracic vertebræ and exposed an almond-shaped tumour, of a dark bluish-red colour, resting upon and attached to the left fourth thoracic (dorsal) nerve, jammed between the dura mater and the spinal cord. The growth was subsequently examined under the microscope, and was found to be composed of fibrous and mucous constituents, quite innocent in character.

Sir Victor Horsley records the fact that a year later the patient was doing his full day's work, which necessitated much walking and standing about.

This classical operation gave the first impetus of an effective character to the development of spinal surgery, and as Mr. Sargent remarks, "There is no single landmark in surgical history which stands out with greater distinctness."

